# Public Awareness, Usage, and Predictors for the Use of Doctor Rating Websites: Cross-Sectional Study in England

**DOI:** 10.2196/jmir.9523

**Published:** 2018-07-25

**Authors:** Salma Patel, Rebecca Cain, Kevin Neailey, Lucy Hooberman

**Affiliations:** ^1^ School of Health and Society University of Salford Manchester United Kingdom; ^2^ Loughborough Design School Loughborough University Loughborough United Kingdom; ^3^ Warwick Manufacturing Group University of Warwick Coventry United Kingdom

**Keywords:** online reviews, Physician quality, primary care, Internet, quality patient empowerment, quality transparency, public reporting

## Abstract

**Background:**

With the advent and popularity of social media and consumer rating websites, as well as the emergence of the digitally engaged patient, there has been an increased interest in doctor rating websites or online patient feedback websites, both inside and outside academia. However, there is very little known about how the public across England views such rating websites as a mode to give patient experience feedback.

**Objective:**

The aim of the overall study was to measure and understand public awareness, usage, and attitudes towards doctor rating websites as a mode to give experiential feedback about GPs in general practice in England. This paper reports on the findings of one of the aims of the study, which was to measure public awareness, current usage and future consideration of usage of online patient feedback websites, within the context of other feedback methods, This could allow the value of online patient feedback websites to be determined from the patients’ perspective.

**Methods:**

A mixed methods population questionnaire was designed, validated and implemented face-to-face using a cross-sectional design with a representative sample of the public (n=844) in England. The results of the questionnaire were analyzed using chi-square tests, binomial logistic regressions, and content analysis. The qualitative results will be reported elsewhere.

**Results:**

Public awareness of online patient feedback websites as a channel to leave experiential feedback about GPs was found to be low at 15.2% (128/844). However, usage and future consideration to use online patient feedback websites were found to be extremely low, with current patient usage at just 0.4% (3/844), and patient intention to use online patient feedback in the future at 17.8% (150/844). Furthermore, only 4.0-5.0% of those who would consider leaving feedback about a GP in the future selected doctor rating websites as their most preferred method; more than half of patients said they would consider leaving feedback about GPs using another method, but not using an online patient feedback website.

**Conclusions:**

The findings suggest that online patient feedback websites may not be an effective channel for collecting feedback on patient experience in general practice. Feedback on online patient feedback websites is not likely to be representative of the patient experience in the near future, challenging the use of online patient feedback not just as a mode for collecting patient experience data, but for patient choice and monitoring too. We recommend the National Health Service channels its investment and resources towards providing more direct and private feedback methods in general practice (such as opportunities for face-to-face feedback, email-based feedback, and web-based private feedback forms), as these are currently much more likely to be used by the majority of patients in England.

## Introduction

Since the 1990s, there has been an exponential increase in the usage of the internet around the world, including a rise in the number of people using the internet for health purposes [1]. There has also been a growth in the number of people giving ratings and reviews online for products and services (such as on amazon.com). Some argue that this has allowed for transparent information and communication to influence change and has provided opportunities for consumers to read reviews and make more informed choices [2-4]

The National Health Service (NHS) when founded in 1948 was paternalistic in its approach to the care of patients [5]. However, from the 1970s onwards, there has been an increasing emphasis on patient and public involvement (PPI), with the introduction of multiple measures to collect patient experience feedback, and the provision of more patient choice [5-7]. There has also been a growing emphasis on public reporting of performance measures across the government, including healthcare. Patients are now argued to have an equal relationship with the NHS and other healthcare providers [5,8].

All of the above factors led to the evolution of online patient feedback (OPF) websites or doctor-rating websites. NHS England introduced an OPF website in 2007—the NHS Choices feedback website [9]. For primary care and general practice, this means that patients can use these types of websites to review their healthcare experience and use these reviews to choose a provider. The presence of these websites has been argued to yield multiple benefits, including empowering patients, improving transparency and enhancing patient choice [9-11]. However, there is little evidence to support these claims.

Despite this, there has been a growth in the volume of OPF, which may suggest that patients in England (and other parts of the world) are embracing the opportunity to review their health care online [11-14]. Similarly, growth in the development of OPF can also be seen, with the development of websites where patients can review their medication and treatment plan [15].

There has also been a steady increase in research into OPF websites, with studies conducted in the UK [9,11,13,14,16--24], Germany [16-21], Netherlands [22], Australia [23] and other countries [24,25] all contributing to the OPF evidence basis. Some evidence can be found to suggest that there is an association between online ratings and the quality of care [12,13,26-28], but the results are often conflicting [29].

Studies conducted outside of England have focused on the characteristics of patients that use OPF websites [18,30-33]. However, the findings cannot be directly applied to England due to the nature of the healthcare systems being distinctly different [10]. Furthermore, the main OPF website in England is a practice-based OPF website, where patients leave reviews under a practice name, rather than the name of the general practitioner (GP).

In England, 3 studies focused on OPF websites from the patients’ perspective [34-36]. The first is a qualitative study based on 3 focus groups conducted by the Nuffield Trust which explored public attitudes towards health and social care ratings. The findings suggested that patients relied more on the word-of-mouth to choose a GP rather than an overall score of a GP [34].

The second was a small convenience survey study conducted with 200 participants in one borough of London [35] to explore the predictors for the usage of doctor-rating websites. The findings suggested a low awareness of doctor-rating websites. Those younger, or ethnically white, or those when deciding where to receive care either give importance to the reputation of the doctor or hospital statistics, are more likely to be aware of doctor-rating websites. They also found that income, ethnicity, and the doctor-patient relationship were significant predictors of future intention to use doctor-rating websites.

This latter study was small and was not representative of patients across England. More crucially, however, it was not evident from the study for which purpose patients were using or were aware of these websites (for feedback or choice or both). Furthermore, none of the studies found in the literature compared patient awareness, usage or predictors of OPF to other methods of collecting feedback that are available for patients to use. This means that it is difficult to truly determine usage or awareness outside of its context. Hence, for example, it may be that usage of other methods is also low too, and therefore limited usage is not exclusive to OPF websites. It is also not clear whether OPF is filling a feedback gap”.

The authors of this paper, therefore, conducted a small qualitative study (n=18) to explore patients’ views towards giving online feedback and ratings to GPs in England. This was done within the context of other feedback methods available in primary care, in particular, paper-based feedback cards, which has been published [36]. This current study is a follow up to that study [36], to explore nationwide public views towards online patient feedback or feedback on doctor-rating websites (both terms are used interchangeably in this paper) in England.

The aim of this study was to measure and understand public awareness, usage, and attitudes towards doctor-rating websites, within the context of other feedback methods. Understanding how patients perceive and use OPF websites in comparison to other feedback methods can help determine whether OPF websites are of any perceived value to patients. This may potentially even help increase usage of OPF websites and improve the design and user-experience of OPF websites. This also allows for adequate comparison and a more comprehensive understanding of public awareness and usage of doctor-rating websites, rather than an isolated one, as previous researchers in this field have conducted [18,31,32,35,37]. These researchers also explored the effect or association of socio-demographic variables and other health factors on the usage and awareness of doctor-rating websites and used some of the factors to explain the variation in results. This was also conducted in this study.

This study was also unique in that it focused specifically on using doctor-rating websites to give feedback about GPs, whereas all of the previous studies [18,31,32,35,37] explored doctor-rating websites more generally (for feedback and choice), and asked respondents to comment on its overall use for all healthcare services.

This paper addresses the research question: Are patients aware of OPF websites as a channel for experiential feedback in general practice, and do they use them? (The other mainly qualitative findings of this study will be reported elsewhere).

## Methods

### Questionnaire Design and Mode

A mixed methods population questionnaire was developed by the first author (SP) using the themes that emerged from the authors’ qualitative study [36] and previous literature (see Multimedia Appendix 1 for a copy). It was evaluated and validated based on the Total Survey Error Framework [38] using 7 stages, which included multiple-stage expert reviews (n=16), cognitive interviews (n=9), and pilot testing (n=22). The study had ethical approval from the Biomedical and Scientific Research Ethics Committee at the University of Warwick (ref REGO-2015-1472; May 2015 and #REGO-2015-1472 AM01; Dec 2015).

A decision was made for Ipsos Mori (a research company) to implement the questionnaire face-to-face with a representative sample of the public across England. Face-to-face was the most appropriate mode because of the length of the questionnaire, it was within budget, and it is also least burdensome on the respondent [39]. Ipsos MORI was chosen because they are a reputable and well-experienced research company, who also conducts the national GP Patient Survey on behalf of NHS England (and the Department of Health).

### Sample Size and Sampling Procedure

An target sample size of 850 members of the public (in England) was set based on guidance from Field [40] to allow prevalence statistical estimate proportions to be within 3.5% confidence interval with 95% confidence level. A post-hoc sample size analysis illustrated that the prevalence data was within a confidence interval of 3.37% with a 95% confidence level.

Random location quota sampling using quotas for age, working status, gender and tenure within the region were used in this study. There were 2 stages to the sampling. In the first stage of sampling, approximately 180 Local Area Authorities were randomly selected from all those in the UK, some of which were in Scotland and Wales and therefore do not feature in this study. In the second stage of sampling, one Output Area (a small area made up of around 60 to a 100 addresses) was randomly selected from each of the Local Area Authorities selected in the first stage. These were the output areas where interviewers went to conduct the interviews with the public. Interviewers (n=155) were given quotas of people to interview for each Output Area according to age, working status, gender and tenure within the region.

### Data Collection Procedure

The questionnaire from this study (which was around 10 minutes long) was included in the Ipsos MORI Face-to-Face Omnibus survey called Capibus (which runs every week and is around 30 minutes long) and was conducted using the Computer Assisted Personal Interviewing technique (ie, face-to-face interviews assisted by a computer) by 155 trained interviewers in people’s homes from January 29, 2016 to February 10, 2016. Informed consent was taken verbally from all respondents before entering their homes. Interviewers went door to door and invited the person who answered to take part. The visits were spread out during the week, including evenings and weekends.

During the interview, interviewers immediately noted down each response on to their laptops, and the results were collated in real-time and recorded centrally by Ipsos MORI. There were 110/844 (13%) of all interviews validated (back-checked) so that the interview data was validated according to the ISO 20252 guide

### Data Preparation

The data captured was provided to the first author (SP) in an SPSS file and Excel files. There was no missing data because the computer programming of the script ensured all respondents answered the relevant questions.

#### Weighting the Quantitative Survey Data

The sample profile produced for this study was similar to that achieved on The National Readership Survey (NRS), which uses random probability sampling. Therefore, using rim-weighting, only a very small corrective weighting was applied (on gender, age, social grade, region, working status, tenure, and ethnicity) by Ipsos MORI to adjust the final results to make them in-line with the national demographic profile. This was so that any minor deficiencies or biases in the sample could be corrected and to ensure that the sample was as close to a nationally representative sample.

The unweighted and weighted profile data can be seen in Table 1, which shows minor differences between profiles. For the responses to the questions on the questionnaire, the overall responses between the weighted and unweighted data varied if at all by only 1% or 2%.

### Data Analysis

IBM SPSS Statistics 22 was used to conduct the statistical analysis (content analysis was conducted on the qualitative data, and the results for which will be reported elsewhere). The sampling weights provided by Ipsos MORI were first applied to the data to correct for known sample biases. Univariate analysis or descriptive statistics was performed to describe respondent demographics, and responses to all other relevant questions.

Bivariate analysis was used to describe differences for the main variables (dependent variables, for example, awareness, usage) with the demographic characteristics (independent variables, for example, gender and age). All variables were categorical, and therefore a 2-tailed chi-square test (or Pearson’s test where appropriate) was used, with <.05 considered to be statistically significant. The demographic independent variables (eg, gender and age) were then included in binomial logistic regression models, which were adjusted manually to determine which demographic factors in combination had a signification association or were predictors for the dependent variable [41]. Results were presented as odds ratio and 95% confidence intervals, using the format recommended by Peacock and Kerry [42] for publication. The results for the first binomial logistic regression model and its interpretation were checked and approved by an experienced academic medical statistician in March 2016.

## Results

### Response Rate and Demographic Characteristics

A total of 844 respondents over the age of 15 years from England responded to the questionnaire. The sociodemographics that respondents were asked about included gender, age, social grade, region, qualification, income and ethnicity, and these are reported in Table 1, including both the weighted data used in the analysis as well as the unweighted data. There were 4 further questions related to internet usage and health also asked, and the responses to these are also listed in Table 1. These 11 demographic variables are the independent variables against which other dependent variables were checked for association during the analysis. Further details are in the forthcoming sections.

### Results on Awareness

#### Awareness of the Opportunity to Give Feedback About Care From General Practitioners Using Any Method

A total of 326 of 844 (38.6%) respondents were aware that they could give feedback about their experience of receiving care from a GP, whereas 518 (61.4%) were not aware that they could give feedback at all.

The effect of 11 demographic variables (in Table 1) on awareness was explored using chi-square tests and binomial logistic regression. The following 4 variables (Textbox 1) remained significant (also see Table 2).

#### Awareness of Doctor-Rating Websites for Giving Feedback About Experience of Receiving Care From General Practitioners

All respondents were provided an explanation of doctor-rating websites on screen and verbally by the interviewer (see Multimedia Appendix 1). They were then asked if they had been aware of doctor-rating websites before this survey. A total of 128 of 844 (15.2%) of respondents said that they had been aware of doctor-rating websites previously, and 716 (84.8%) said they had not.

The effect of 11 demographic variables (in Table 1) as well as 2 other relevant variables (1) being aware of the option to give feedback in general about GPs, and (2) having given feedback about GPs in the past were explored on the awareness of doctor-rating websites using chi-square tests and binomial logistic regression. The following 3 variables (Textbox 2) were found to be significant (see Table 3).

Qualifications and income were predictors for the awareness of the option to leave feedback using any method but were not found to be predictors for the awareness of doctor-rating websites.

#### Which, If Any, of the Following Doctor-Rating Websites Are You Aware Of?

From the 128 of 844 (15.2%) respondents who were aware of doctor-rating websites, 54/128 (42.2%) said they were not aware of a specific website. In total, 61/128 (47.7%) were aware of NHS Choices feedback site, 20/128 (15.6%) were aware of Patient Opinion, 5/128 (3.9%) were aware of PrivateHealth, 1/128 (0.8%) were aware of iwantgreatcare, and 2/128 (1.6%) mentioned “other.” This means that from all the respondents, only 61/844 (7.2%) were aware of the NHS Choices feedback site, and 20/844 (2.4%) were aware of Patient Opinion.

### Results on Past Usage of Online Rating Websites

#### Past Experience of Giving Feedback About General Practitioners Using Any Method

There were 161 of 844 (19.1%) respondents that said they had formally given feedback about the care they had received from a GP in the past, and 683/844 (80.9%) said they had not. Of those who had given feedback formally in the past, 94/161 (58.4%) had given it directly to the GP, and 57/161 (35.4%) had given it to the GP practice. The remaining 10/161 (6.2%) had given it to an external organization.

The effect of 11 demographic variables (Table 1) on whether someone had given feedback in the past about their experience of receiving care from a GP was explored using chi-square tests and binomial logistic regression. There were 2 variables (Textbox 3) found to be significant (also see Table 4).

#### Past Usage of Doctor-Rating Websites for Any Purpose

Respondents who were aware of doctor-rating websites were asked if they had used a doctor-rating website before. Nineteen out of 128 (14.8%) had done so in the past, and the remaining 108/128 (84.4%) had not. This means that in total, from all the respondents, only 15/844 (1.8%) had used a doctor-rating website before. Given the amount the NHS and other external organizations have invested in establishing OPF websites, and the popularity of other rating websites like TripAdvisor, the very low level of usage at 15 is surprising.

The effect of 11 demographic variables (in Table 1) on the usage of doctor-rating websites was explored using Fisher’s exact test and binomial logistic regression. The variables ethnic origin (*P*=.043) and region (*P*=.041) as well as having searched the internet for health information previously (*P*=.007) were found using Fisher’s exact test to be significant on the usage of doctor-rating websites. The combined effect of all variables was investigated using binomial logistic regression; however, none of the variables were found to be significant (*P*>.05). Thus, it would seem that while having searched for health information in the past was found to be a predictor for the awareness of doctor-rating website and future consideration of using doctor-rating websites; it is not a predictor for usage.

**Table 1:** The 11 demographic characteristics of the respondents of the questionnaire (n=844).

Demographic characteristics		Respondents, n (%)		Difference between unweighted and weighted data, %
		Unweighted data	Weighted data	
**Gender**				
	Male	433 (51.3)	413 (48.9)	–2.4
	Female	411 (48.7)	431 (51.1)	+2.4
**Age (years)**				
	15-24	150 (17.8)	132 (15.7)	–2.1
	25-34	112 (13.3)	142 (16.8)	+3.5
	35-44	116 (13.7)	134 (15.9)	+2.2
	45-54	138 (16.4)	144 (17.1)	+0.7
	55-59	58 (6.9)	51 (6.1)	–0.8
	60-64	67 (7.9)	63 (7.4)	–0.5
	65+	203 (24.1)	178 (21.0)	–3.0
**Social grade^a^**				
	AB	191 (22.6)	231 (27.4)	+4.8
	C1/C2	435 (51.5)	412 (48.8)	–2.7
	D	124 (14.7)	129 (15.3)	+0.6
	E	94 (11.1)	72 (8.6)	+2.5
**Government office region**				
	East Midlands	56 (6.6)	73 (8.6)	+2.0
	Eastern	71 (8.4)	94 (11.1)	+2.7
	London	137 (16.2)	130 (15.5)	–0.7
	North East	41 (4.9)	41 (4.9)	0.0
	North West	126 (14.9)	111 (13.2)	–1.7
	South East	111 (13.2)	137 (16.3)	+3.1
	South West	100 (11.8)	86 (10.2)	–1.6
	West Midlands	101 (12.0)	88 (10.4)	–1.6
	Yorkshire and Humber	101 (12.0)	84 (9.9)	–2.1
**Qualification**				
	GCSE/ O-LV/CSE/NVQ12^b^	215 (25.5)	212 (25.1)	–0.4
	A-level or equivalent	168 (19.9)	160 (18.9)	–1.0
	Bachelor/Master/PhD	234 (27.7)	264 (31.3)	+3.6
	No formal qualification	168 (19.9)	150 (17.8)	–2.1
	Other	59 (7.0)	59 (7.0)	0.0
**Income (£)**				
	<11,499	102 (12.1)	88 (10.4)	–1.7
	11,500-17,499	78 (9.2)	76 (9.0)	–0.2
	17,500-24,999	47 (5.6)	45 (5.4)	–0.2
	25,000-29,999	56 (6.6)	54 (6.4)	–0.2
	30,000-39,999	63 (7.5)	68 (8.0)	+0.5
	40,000-49,999	49 (5.8)	54 (6.4)	+0.6
	50,000-74,999	66 (7.8)	86 (10.2)	+2.4
	>75,000	35 (4.1)	44 (5.3)	+1.2
	Don't know	158 (18.7)	153 (18.2)	–0.5
	Refused	190 (22.5)	176 (20.8)	–1.7
**Ethnicity**				
	White	710 (84.1)	723 (85.9)	+1.8
	Non-white	134 (15.9)	118 (14.1)	–1.8
**Internet access frequency**				
	Daily	657 (77.8)	679 (80.4)	+2.6
	Weekly	67 (7.9)	62 (7.3)	–0.6
	Monthly	14 (1.7)	12 (1.5)	–0.2
	Never	106 (12.6)	91 (10.8)	–1.8
**Have you ever used the internet to search for health information?**				
	Yes	434 (51.4)	458 (54.2)	+2.8
	No	410 (48.6)	386 (45.8)	–2.8
**Do you have a long-term health condition?**				
	Yes	241 (28.6)	222 (26.3)	–2.3
	No	603 (71.4)	622 (73.7)	+2.3
**Approximately how many General Practitioners are there in your current general practitioner surgery?**				
	1	31 (3.7)	29 (3.5)	–0.2
	2-3	203 (24.1)	197 (23.3)	–0.8
	4-5	265 (31.4)	268 (31.8)	+0.4
	6-9	206 (24.4)	210 (24.9)	+0.5
	>10	45 (5.3)	45 (5.3)	0.0
	Don’t know	94 (11.1)	95 (11.2)	+0.1

^a^A: Higher managerial, administrative and professional; B: Intermediate managerial, administrative and professional; C1: Supervisory, clerical and junior managerial, administrative and professional; C2: Skilled manual workers; D: Semi-skilled and unskilled manual workers; E: State pensioners, casual and lowest grade workers, unemployed with state benefits only.

^b^GCSE: General Certificate of Secondary Education; O-LV: General Certificate of Education: Ordinary Level; CSE: Certificate of Secondary Education; NVQ: National Vocational Qualification.

**The 4 significant variables.**

1. Income (£): This was found to be statistically significant (*P*=.003), and those with an income of £50,000-£74,999 had the highest odds and were 2.2 times more likely to be aware of the option to give feedback about their experience of care from a general practitioner (GP), in comparison to those whose income was below £11,499.
2. Qualification: This was found to be statistically significant (*P*=.002), and those with a graduate qualification had the highest odds and were also 2.2 times more likely to be aware than those with no formal qualifications.
3. The presence or absence of a long-term condition: This was found to be statistically significant (*P*=.004), and those who did have a long-term condition were 1.6 times more likely to be aware of the option to give feedback about a GP than those who did not have a long-term condition.
4. The number of GPs in the respondents’ surgery: This was also found to be statistically significant (*P*=.02), with those who were not aware of the number of GPs present in their surgery being the least likely (64.4%) to be aware of the option to give feedback about GPs, as compared with those who were aware that they had 1 GP in their surgery.

**Table 2:** Odds ratio adjusted for all the other variables for the effect of set demographic variables on the awareness of the option to give feedback about a general practitioner (n=844). The term “Ref” refers to the reference category (odds ratio of 1.000).

Variable		Odds ratio		95% CI	
**Income (£)^a^**					
	<11,499		Ref (1.000)		—
	11,500-17,499		1.790		0.937-3.420
	17,500-24,999		1.303		0.608-2.792
	25,000-29,999		1.126		0.547-2.317
	30,000-39,999		1.307		0.660-2.591
	40,000-49,999		0.892		0.425-1.872
	50,000-74,999^b^		2.211		1.131-4.320
	>75,000		0.534		0.234-1.219
	Don't know		0.789		0.436-1.429
	Refused		0.826		0.472-1.445
**Qualification^c^**					
	No formal qualification		Ref (1.000)		—
	GCSE/O-level/CSE/NVQ^d^		1.020		0.628-1.659
	A-level or equivalent		1.386		0.832-2.309
	Degree/masters/PhD or equivalent^b^		2.197		1.350-3.575
	Other		1.463		0.761-2.811
**Long-term condition^e^**					
	No		Ref (1.000)		—
	Yes^b^		1.631		1.166-2.283
**Number of General Practitioners in the surgery^f^**					
	1		Ref (1.000)		—
	2-3		0.902		0.389-2.090
	4-5		0.899		0.392-2.065
	6-9		0.867		0.372-2.018
	>10		0.479		0.170-1.352
	Don’t know^b^		0.356		0.138-0.917

^a^*P*=.003

^b^*P*=.05

^c^*P*=.002

^d^GCSE: General Certificate of Secondary Education; O-LV: General Certificate of Education: Ordinary Level; CSE: Certificate of Secondary Education; NVQ: National Vocational Qualification.

^e^*P*=.004

^f^*P*=.019

**The 3 significant variables.**

1. Age: This was found to be significant (*P*=.02), with those between the ages of 60-64 being 63% less likely to be aware of doctor-rating websites than those aged 35-44.
2. Those who had searched for health information on the internet in the past were 2.7 times more likely to be aware of doctor-rating websites than those who had not.
3. Also, those who were aware of the option to give feedback about a general practitioner using any method, were 5.6 times more likely to be aware of the existence of doctor-rating. websites than those who were not aware, suggesting that being aware of any method of giving feedback is a predictor for awareness of doctor-rating websites.

**Table 3:** Adjusted odds ratio for the effect of a set of demographics and 2 other variables on whether someone was aware of doctor-rating websites. Each odds ratio is adjusted for all the other variables in the table (n=844). The term “Ref” refers to reference category (odds ratio of 1.000).

Variable		Odds ratio	95% CI
**Age (years)^a^**			
	15-24	0.425	0.181-1.000
	25-34	1.442	0.753-2.762
	35–44	Ref (1.000)	—
	45-54	0.974	0.493-1.927
	55–59	1.473	0.627-3.461
	60–64^b^	0.366	0.127-1.057
	>65	0.779	0.399-1.523
**Past use of internet to search for health information^c^**			
	No	Ref (1.000)	—
	Yes^b^	2.690	1.709-4.234
**Awareness of the option to give feedback about general practitioners^c^**			
	No	Ref (1.000)	—
	Yes^b^	5.632	3.631-8.737

^a^*P*=.02

^b^*P*=.05

^b^*P*<.001

**The 2 significant variables.**

1. Gender was found to be statistically significant (*P*=.002), with female respondents almost twice as likely to have given feedback in the past than male respondents.
2. The presence or absence of a long-term health condition was found to be significant (*P*=.002), with those with a long-term health condition 1.8 times more likely to have given feedback about their experience of receiving care from a general practitioner in the past.

**Table 4:** Odds ratio adjusted for all the other variables in the table for the effect of a set of demographics on whether someone had given feedback about their experience of receiving care from a general practitioner in the past (n=844). The term “Ref” refers to reference category (odds ratio of 1.000).

Variable		Odds ratio	95% CI
**Gender^a^**			
	Female	Ref (1.000)	—
	Male	0.574	0.403-0.819
**Long-term health condition**			
	No	Ref (1.000)	—
	Yes	1.782	1.233-2.576

^a^*P*=.002

**The 7 significant variables.**

1. Gender: This was found to be statistically significant (*P*=.01), with male respondents less likely to consider giving feedback in the future than females.
2. Age: This was also found to be statistically significant (*P*=.001), with those aged between 35-44, 55-59, and 60-64 around 2.5 times more likely to consider leaving feedback than those aged >65.
3. Long-term health condition: These were also twice more likely to consider leaving feedback than those did not have a health condition, as may be expected.
4. Who had used the internet in the past to search for health information: These were more than twice as likely to consider leaving feedback in the future than those who had not used the internet in the past to search for health information.
5. Number of general practitioners (GPs) in the respondents’ surgery: This was also found to be significant with those who had 2-3 GPs in their surgery found to be 2.5 times more likely to consider leaving feedback than those who had just 1 GP in their surgery.
6. Qualification: This was also found to be statistically significant (*P*<.001), with those who had a graduate qualification being 4 times more likely to consider leaving feedback than those with no qualifications, and those with GCSEs or equivalent twice as likely to leave feedback than those with no qualifications.
7. Region: This was also found to be significant (*P*<.001), with those living in the North West, South East and Yorkshire and Humber, twice as likely to consider leaving feedback than those living in London, and those living in the North East 4.8 times more likely to consider leaving feedback than those living in London.

#### Past Usage of Doctor-Rating Websites for Giving Feedback About a General Practitioner

From the 19 respondents who had used a doctor-rating site before, 8/19 (42.1%) had used it to read a review for a doctor or hospital, 5/19 (26.3%) had used it to find a doctor or hospital, 4/19 (21.1%) had used it to review their experience of the NHS, and 3/19 (15.8%) had used it to give feedback about their experience of receiving care from a GP. Therefore, only 3/844 (0.4%) of the entire sample of respondents had used a doctor-rating website in the past to give feedback about their experience of receiving care from a GP.

From the 3 participants that left feedback on a doctor-rating website about a GP, 2 commented on a positive experience, and 1 commented on a negative experience. The reasons the 3 respondents gave for leaving feedback online was that they either wanted to let the GP know how much they appreciated the consultation or they believed sharing their experience would benefit the GP, or they wanted to comment on their treatment in general. No other reasons were cited.

### Future Use of Online Rating Websites

#### Consideration of Giving Feedback in the Future Using Any Method

All respondents were asked whether they would consider giving feedback in the future about their experience of receiving care from a GP. A total of 638 of 844 (75.6%) respondents said they would consider giving feedback in the future, 214 (25.4%) said definitely, and 424 (50.0%) said possibly. A total of 199 (23.6%) said they would not consider giving feedback in the future, and 7 (0.8%) said they do not know.

Responses were first combined to form a bivariate variable of yes and no. The effect of 11 demographic variables (in Table 1) on consideration of giving feedback in the future was then explored using chi-square tests and binomial logistic regression. Seven variables were found to be statistically significant (Textbox 4 and Table 5).

#### Consideration of Future Use of Doctor-Rating Websites to Give Feedback About General Practitioners

A total of 18 of 844 (2.1%) respondents said they would consider using doctor-rating websites to give feedback about their experience of care from a GP (ie, a GP who is based in a surgery).

The effects of the 11 demographic variables (in Table 1) on the consideration of future use of doctor-rating websites was explored as well as the following additional variables: (1) awareness of doctor-rating websites, (2) past use of doctor-rating websites, (3) consideration of future use of doctor-rating websites for any purpose, and (4) consideration of giving feedback in the future about a GP. After using chi-square tests and binomial logistic regression, only past use of internet to search for health information remained significant (*P*=.007; please see Textbox 5 and Table 6).

#### Public Preference on Mode of Feedback

All respondents who said they would consider giving feedback in the future about a GP (n=776) were asked which mode they would most prefer using to give feedback about their experience with a GP, for both negative and positive feedback. They were provided with a list of 15 methods and were first asked to select the top 3 most preferred ways (or modes) to leave feedback and then their main preference. The complete sets of results are provided in Multimedia Appendix 2.

**Figure 1:**
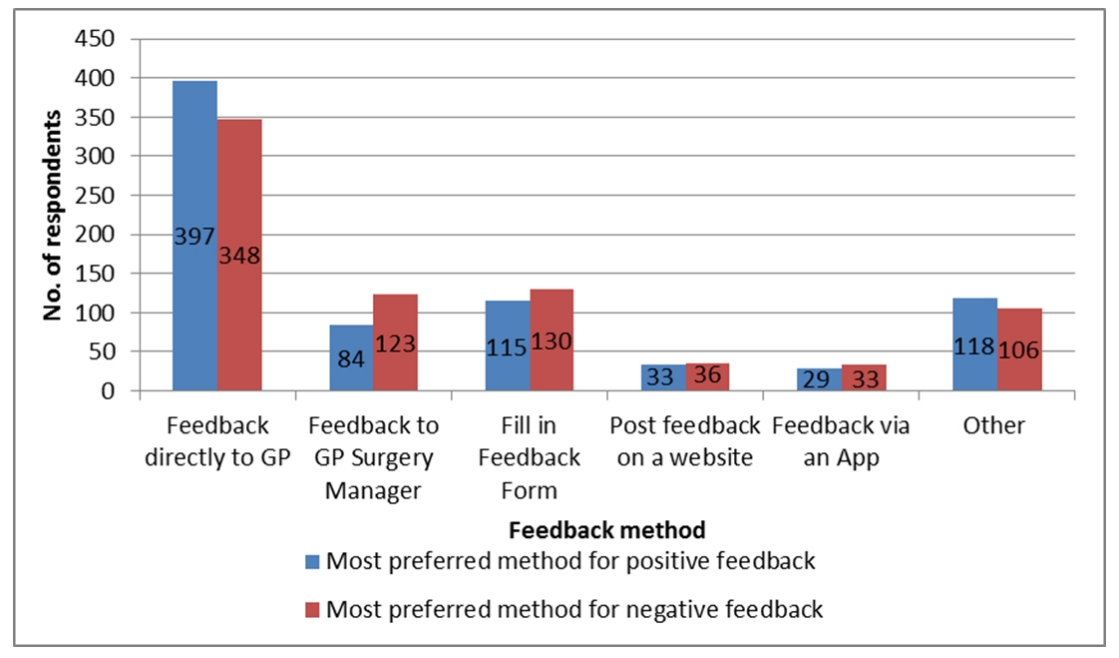
Summary of respondents' (n=776) main preference for giving feedback about their experience of receiving care from a general practitioner (GP).

In summary (see Figure 1), the main preferences of respondents for giving feedback about their experience with a GP was (1) giving feedback directly to the GP where 397/776 (51.2%) selected this for positive feedback, and 348 (44.8%) for negative feedback, (2) giving feedback to the GP surgery manager where 84 (10.8%) for positive, and 123 (15.9%) for negative, (3) filling in a feedback form at the surgery or on the practice’s website where 115 (14.8%) for positive, and 130 (16.8%) for negative, (4) posting feedback on a public website where 33 (4.3%) for positive, 36 (4.6%) for negative, and (5) giving feedback through an app where 29 (3.7%) for positive, and 33 (4.3%) for negative.

**Table 5:** Adjusted odds ratio for all the other variables in the table for the effect of a set of demographics on whether someone will consider giving feedback in the future about a general practitioner (n=844). The term “Ref” refers to reference category (odds ratio of 1.000).

Variable		Odds ratio	95% CI
**Gender^a^**			
	Female	Ref (1.000)	—
	Male^b^	0.630	0.438-0.906
**Age (years)^c^**			
	15-24	0.866	0.457-1.638
	25-34	1.607	0.833-3.102
	35-44^b^	2.617	1.328-5.156
	45-54	0.864	0.475-1.570
	55-59^b^	2.555	0.992-6.578
	60-64^b^	2.483	1.071-5.754
	>65	Ref (1.000)	—
**Region^c^**			
	London	Ref (1.000)	—
	East Midlands	0.584	0.284-1.200
	Eastern^b^	0.825	0.428-1.590
	North East^b^	4.823	1.489-15.628
	North West^b^	2.330	1.167-4.649
	South East^b^	2.448	1.178-5.084
	South West	2.298	1.055-5.003
	West Midlands	0.979	0.512-1.870
	Yorks and Humber^b^	2.357	1.093-5.082
**Qualifications^c^**			
	No Formal Qualifications	Ref (1.000)	—
	GCSE/O-Level/CSE/NVQ^d^	2.126	1.238-3.650
	A-Level or Equivalent (=NVQ3)	1.714	0.952-3.084
	Bachelors/Masters/PhD Or Equivalent^b^	4.086	2.287-7.298
	Other^b^	2.649	1.166-6.019
**Past use of internet to search for health information^c^**			
	No	Ref (1.000)	—
	Yes^b^	2.392	1.624-3.524
**Long-term health condition^e^**			
	No	Ref (1.000)	—
	Yes^b^	2.078 (1.000)	1.257-3.433
**No. of General Practitioners in surgery^f^**			
	1	Ref (1.000)	—
	2-3^b^	2.511	1.034-6.097
	4-5	2.010	0.823-4.911
	6-9	2.275	0.894-5.794
	>10	2.318	0.648-8.286
	Don't know	0.759	0.292-1.975

^a^*P*=.013

^b^*P*=.05

^c^*P*<.001

^d^GCSE: General Certificate of Secondary Education; O-LV: General Certificate of Education: Ordinary Level; CSE: Certificate of Secondary Education; NVQ: National Vocational Qualification.

^e^*P*=.004

^f^*P*=.002

**The 1 significant variable after logistic regression.**

Only past use of internet to search for health information remained significant (*P*=.007), with those who had used the internet to search for health information in the past being 1.6 times more likely to consider using doctor-rating websites to give feedback about a general practitioner (GP), than those who had not previously used the internet to search for health information. This suggests that existing engagement and interest in health, as well as being an indicator for patient awareness of doctor-rating websites (as mentioned earlier), is also an indicator for patient intention to use doctor-rating websites in the future to give feedback about GPs.

**Table 6:** Logistic regression (odds ratio) showing the effect of past use of the internet to search for health information on whether someone would consider using a doctor-rating website to give feedback about a GP (n=844). The term “Ref” refers to reference category (odds ratio of 1.000).

Variable		Odds ratio	95% CI
**Internet to search for health information^a^**			
	No	Ref (1.000)	—
	Yes^b^	1.649	1.144-2.376

^a^*P*=.007

^b^*P*=.05

**Table 7:** Summary of key results from all respondents (n=844) relating to awareness, past usage and future consideration of giving feedback about experience with receiving care from a general practitioner (GP). Additional details are presented in the subsections.

Characteristic	Awareness		Past Use			Future Consideration		
	Any method	Doctor-rating websites		Any method	Doctor-rating websites		Any method	Doctor-rating websites
Positive (yes), n (%)	329 (39.0)	126 (15.0)		160 (19.0)	3 (0.4)		641 (75.9)	151 (17.9)
Gender	—	—		Y^a^	—		Y	—
Age	—	Y		—	—		Y	—
Social grade	—	—		—	—		—	—
Region	—	—		—	—		Y	—
Qualification	Y	—		—	—		Y	—
Income	Y	—		—	—		—	—
Ethnicity	—	—		—	—		—	—
Internet access frequency	—	—		—	—		—	—
Past use of internet to search for health information	—	Y		—	—		Y	Y
Presence or absence of a long-term health condition	Y	—		Y	—		Y	—
Number of GPs in surgery per local health center	Y	—		—	—		Y	—

^a^Y: significant using binomial logistic regression.

It is interesting to note that although results in the previous sub-section indicate that 150/844 (17.0%) of all respondents would consider using doctor-rating websites (both NHS and independent websites) in the future to give feedback about GPs. Only 36 respondents selected a doctor-rating website as their most preferred method to leave negative feedback about GPs. This corresponds to 36/776 (4.6%) of all those who would consider giving feedback, and 36/844 (4.3%) of all respondents. The overwhelming preference for leaving feedback with GPs or the GP surgery correlates with earlier results that indicated that 151/161 (93.8%) of those who had left feedback for or about a GP in the past, had left it with the GP or GP practice.

Figure 1 also demonstrates that patients’ most preferred method to give feedback varies depending on whether their feedback is about a negative or a positive experience. This suggests “patient feedback mode” is partially dependent upon the nature of the experience.

### Summary of the Results

Table 7 summarizes the key results found in this study, as well as the demographic factors that were found to be significant on each of the key dependent variables. The table demonstrates that 128 of 844 (15.2%) respondents were aware of doctor-rating websites for giving feedback about GPs, in comparison to 326 (38.6%) of respondents who were aware of giving feedback using any of the methods. Similarly, 161 (19.1%) of respondents had given feedback about a GP in the past using any method, whereas only 3 (0.4%) had given feedback about a GP using a doctor-rating website. A total of 638 (75.6%) of respondents said they would consider giving feedback about a GP in the future (using any method); whereas only 150 (17.8%) of respondents said they would consider giving feedback in the future using doctor-rating websites.

## Discussion

### Public Awareness of Doctor-Rating Websites

The results suggest that based on a representative sample of 844 respondents, 15.2% (128/844) of the population in England is aware of the existence of doctor-rating websites to give feedback to a GP, whereas 38.6% (326/844) is aware that they can give feedback using any method. The level of awareness found in this study is in line with findings from a previous study by Galizzi et al [35] who found that 15% of their London-based respondents was aware of the existence of doctor-rating websites, although it was not clear for which purpose they were aware of such websites, and which specific websites they were aware of. However, they suggested that this indicated low awareness amongst the population in England.

The findings from the present study suggest that awareness of doctor-rating websites to give feedback about a GP, compared with awareness of the option to give feedback about a GP using any method, is not low. This is because almost half of those who are aware of the option to give feedback about a GP are aware of the existence of doctor-rating websites (for feedback on GPs). Despite this, 54/128 (42.2%) of those that were aware of doctor-rating websites were not aware of a specific website, only 61/844 (7.2%) respondents were aware of the NHS Choices feedback website, and 20/844 (2.4%) of Patient Opinion. This indicates that awareness of specific doctor-rating websites is low, which is surprising given that the NHS Choices feedback website is an official channel for patients in England to leave feedback about healthcare services (although it is unknown how well if at all, it is promoted to patients and the public).

Higher levels of awareness of doctor-rating websites were found outside of the UK, with the highest found in the USA at 65% by Hanauer et al [31], and in Germany, at 29.3% in 2012 [32] and 32% in 2013 [18]. The higher levels of awareness in comparison to what was found in this study may be partially explained by the higher usage and popularity of private healthcare, the competitive nature of healthcare in both countries, and also what may appear to be a higher usage of internet for health seeking information (reported in one study in Germany at 68% [43] when compared to 54% found in this study). But there could also be a sampling effect, as the aforementioned studies were all conducted using online panel sampling. However, a recent study in Germany that used a cross-sectional random sample survey found awareness at 72.5% [21]

The results from the present study indicate that awareness of doctor-rating websites (unlike awareness of giving feedback to a GP in general), is not dependent on being wealthier, having better qualifications, having a long-term condition (and possibly using GP services more frequently) and knowing how many GPs practice in your surgery. Instead, age and having searched for health information in the past were found to be the only predictors for awareness of doctor-rating websites. Age was also found to be significant by Galizzi et al [35], and this they suggest is not surprising because elderly people use the internet less frequently. If a person has searched for health information in the past, this may suggest that: (1) they know how to use the internet (and may have access to it too), and (2) they are actively interested in their health. It is therefore not surprising that they are more likely to be aware of doctor-rating websites.

In London, Galizzi et al [35] found that as well as age, ethnicity was significant for awareness, with white respondents less likely to be aware of these websites; however, this was not found in this study. In Germany, Emmert et al [18] found that differences in age group were not statistically significant, and neither was education, employment, internet use, and health status. However, unlike this study, they found that female respondents were more likely to be aware of doctor-rating websites, as well as those widowed, and those with higher health care utilization. In this study, female respondents were found to be more likely than male respondents to have given feedback in the past using any method.

### Public Usage of Doctor-Rating Websites

Based on the present sample, 19.1% (161/844) of the population in England has given feedback in the past using any method, whereas only 0.4% (3/844)of the population have given feedback using doctor-rating websites, which is significantly lower. The level of use of doctor-rating websites to specifically give feedback or review GPs in England had not been explored in previous studies; however, Galizzi et al [35] did explore usage of doctor-rating websites and found that 3% of their Londoners’ sample (n=200) had used doctor-rating websites, although again it was not evident for which purpose. This is similar to the finding of this study that 1.8% (15/844) of the population had used a doctor-rating website before for any of the purposes. The low level of usage indicates that patients are not using doctor-rating websites, especially not to give feedback about GPs. This is surprising given that the NHS recently spent £1.25M piloting a new doctor-rating website called CareConnect [44].

Outside of the UK, usage of doctor-rating websites was found to be much higher. A recent study in the USA (in Rochester Minnesota) reported that 16% of the surveyed sample had used a doctor-rating website, and 3% had used it to give feedback [37]. This was different to another study in the USA, conducted in 2012, which reported general usage at 23%, and found that 6% of parents had left ratings for doctors online [45]. The difference may be partly because the latter study used an online panel as the survey mode, and the former study used a written questionnaire, or that the former was conducted in a single healthcare setting.

Usage of doctor-rating websites was also high in Germany. In 2013, 25% of the population had used a doctor-rating website to search for a doctor, and 11% to leave feedback or ratings [18]. Similarly, Terlutter et al [32] discovered in Germany (in 2012) that 26% of the population had used a doctor-rating website before, although it was not clear for which purpose. More recently, in 2017, researchers found usage of doctor-rating websites in Germany at 43.6% [21] In Austria, researchers conducted an experimental study based on a convenient sample and found that 47% of respondents had used a doctor-rating website, and 6% had used it to leave feedback [46]. The difference in results may be due to regional differences in the diffusion of doctor-rating websites and the adoption of the internet for seeking health information. However, there may also be a sampling effect, because many of the studies outside of the UK (with the exception of the one conducted by Burkle and Keegan [37] and McLennan et al [21]) used an online panel as their sample population. The use of online sampling may have affected results, because those who are online, and had used the internet to search for health information, may be more likely to be aware of and use doctor-rating websites than those that had not, as results from this present study suggest.

In the USA and Germany, academics found various predictors for usage of doctor-rating websites, such as the presence of a long-term condition, advanced education, age, and gender [18,32,37,45]. Predictors for the usage of doctor-rating websites for feedback about GPs could not be computed in this study because only 0.4% (3/844) of respondents had used a doctor-rating website for that purpose. However, the results do indicate however that female respondents and those with long term health conditions are significantly more likely to have given feedback in the past to a GP (using any method). Those with long-term health conditions tend to use GP services more than those who do not have a long-term health condition, and so it is not surprising that they are more likely to leave feedback.

### Future Use of Doctor-Rating Websites

Although the present study suggests that 75.6% (638/844) of the population in England would consider giving feedback in the future to a GP using any method, only 17.8% (150/844) would consider giving feedback in the future to a GP on a doctor-rating website. This suggests that more than half of respondents would consider giving feedback to a GP but not on a doctor-rating website. Similarly, 33.1% (279/844) of the population would consider using doctor-rating websites but not to leave feedback for a GP. This, as well as the 0.4% (3/844) past usage of doctor-rating websites, and only 4.3% (33/776) to 4.7% (36/776) selecting doctor-rating websites as their most preferred feedback method, questions whether doctor-rating websites are wanted or needed by the public for leaving feedback about GPs.

The only significant predictor for the future use of doctor-rating websites for giving feedback about GPs was the past use of the internet to search for health information, with those that had were found to be 1.6 times more likely to consider using doctor-rating websites to give feedback about a GP than those that had not. This predictor is not surprising given it indicates an active interest in one’s health as well as familiarity with the internet. What was surprising was the absence of 6 other predictors which were found to be significant for the future consideration of using any method to leave feedback about GPs. These predictors indicated that those that are either female, younger in age, have a long-term health condition, have higher qualifications, have more GPs in their surgery, or live outside of London are much more likely to consider leaving feedback about a GP using any method. This could be seen as a positive suggestion that doctor-rating websites, unlike other feedback methods, may span across the age, social and regional divide, and appeal to everyone who takes an active interest in their health and is familiar with the internet to pursue that interest. This appears to support Bardach et al’s [26] argument that OPF websites would collect feedback from those patients who would not normally give feedback. For consideration of using doctor-rating websites in the future for any purpose (and not just giving feedback about a GP), in addition to past use of the internet to search for health information, the respondent’s age and internet consumption were also found to be significant predictors. This is in contrast to Galizzi et al’s [35] findings with Londoners in which income, ethnicity, and the doctor-patient relationship were the significant predictors for future intention to use doctor-rating websites for any purpose.

### Public Preference on Mode of Feedback

The results suggest that there is no one most preferred way for patients to leave feedback about a GP, and this was also found by Patel et al However, like the results of Patel et al [47], the present study also found rather surprisingly that almost half of those who would consider leaving feedback for a GP would prefer to give feedback directly to the GP, even when it is negative feedback. Furthermore, the 2 major reasons for choosing 1 mode of feedback over another were ease and convenience, followed by the method being a direct way of giving feedback (and Patel et al [47] found that the latter was so that patient feedback reaches the GP and is used by the GP for improvement purposes). These are interesting findings because currently there is little formal provision in general practice in England to give feedback directly to the GP.

Current formal provisions for leaving feedback about GPs in the NHS also include the NHS Friends and Family Test card, which is a paper-based feedback form that is used in most GP practices in England [48], and the GP Patient Survey [48]. The paper-based feedback form was only selected by 10% of respondents (who would consider leaving feedback in the future) as their most preferred method for leaving negative feedback for a GP. Similarly, use of OPF websites to report a negative experience was selected as the main preference by only 5% of respondents. In contrast, 45% of patients’ most preferred method to leave negative feedback was directly with the practice and 16% directly with the practice manager. The vast majority of patients (94%) who had given feedback in the past had given it directly to the GP or practice.

These results as well as others suggest that current methods available in general practice to leave feedback are on the whole not the most preferred methods for patients. Therefore, GP practices and the NHS need to consider alternative ways and methods to collect feedback. For example, giving patients the option to send feedback through email, which was selected by 12% of respondents (who would consider leaving feedback in the future) as their most preferred method. This also questions the value of OPF websites and questions whether patients in England want or need these types of websites to leave feedback about GPs.

Although preference for leaving feedback online was minimal, one of the interesting findings from the results was that more people prefer to leave feedback online on a private feedback form on the GP surgery website, rather than leaving it on a (public) doctor-rating website. Similarly, although more people preferred to give feedback directly to the GP in person or telephone, in comparison to writing a letter, more respondents preferred to use email to send feedback. Furthermore, an app was found to be almost the same in popularity as leaving feedback on a doctor-rating website, although again the main preference was to use an app that would give the feedback to the GP surgery directly rather than an app that would publish the feedback online. These findings support the notion that many patients prefer to give feedback directly to the GP and practice rather than leaving feedback in the public domain, and these alternative modes of leaving feedback need to be taken into consideration by GPs and GP practices in England, if they want to engage and increase the volume of patient experience feedback.

### Strengths and Limitations

The strength of this study lay in its use of a well-validated mixed methods population questionnaire whose aim was to measure representative views of the public on giving feedback about GPs on OPF websites, within the context of other feedback mechanisms. Nevertheless, this study did have several limitations.

Firstly, the sampling method used—a random location quota sampling—was not a random sample, and although the data was weighted so that it would be a representative sample of the population in England, the sample may still contain biases, and claiming generalizability (external validity) across the whole population in England could be questioned. However, given that it was not feasible to get a random sample of the population in England, this was as close as possible to a true representative sample and very little correction of the results was needed to make them in line with The National Readership Survey (NRS), which uses random probability sampling. The interviews were also conducted face-to-face, which meant that there was very little risk of respondents misunderstanding the questions, and there was a lower risk of premature termination, as interviewers could keep respondents motivated.

Secondly, although the questionnaire had strong internal validity, the fieldwork was conducted by 155 interviewers from Ipsos MORI, and not the authors, and this could be a potential weakness. Nevertheless, the interviewers were all experienced professional interviewers who were trained by Ipsos MORI and given the same very specific instructions. A validation procedure on the fieldwork was also conducted to ensure that interviewers had interviewed respondents as expected.

Thirdly, the results of this study question the value of providing OPF websites in England to give feedback about GPs; however, this study did not explore patients’ views on OPF websites for choice, an issue that was outside the scope of this study. Although both giving feedback and patient choice are highly connected (because if patients do not give feedback or reviews online, other patients will not have these patient reviews to choose from), they are distinctly different as actions. The results when reported in this study make clear that they are specifically about giving feedback on GPs only.

Fourthly, this study focused on primary care and GPs only in England. The results may have been different if the study focused on other healthcare professionals such as surgeons, or secondary care.

### Implications for Practice

The results from this study strongly suggest that GPs, GP practices, the NHS, and feedback website providers should consider alternative mechanisms to collect patient feedback in general practice, instead of relying primarily on the NHS Family and Friends Test card and online patient feedback websites as a day-to-day feedback method. In particular, direct methods to give feedback to the GP or the GP practice (digital or non-digital) are most used and preferred by patients, such as face-to-face feedback, email, telephone, and private feedback forms on the GP practice website. Therefore, we recommend the NHS to channel its investment and resources towards providing more direct and private feedback methods in general practice (such as opportunities for face-to-face feedback, email-based feedback, and Web-based private feedback forms), as these are much more likely to be used currently by the majority of patients in England. We also recommend that when online feedback is presented to other patients for choice”, the feedback must be part of a collection of measures including patient feedback collected using other methods, and other measures such as the clinical competency of the GP, findings from the Care Quality Commission report, and safety results. Other recommendations for OPF providers and GPs and GP practices can be found in Multimedia Appendix 3 and Multimedia Appendix 4 respectively, where we also highlight what the NHS and other OPF providers can do to increase patient use of OPF websites.

### Conclusions

This is the first piece of nationally representative research that has explored patients’ awareness and usage of OPF websites within the context of other feedback mechanisms available in general practice in England, and to date, in our knowledge, the largest and most robust study conducted with patients about doctor-rating websites.

Given the popularity, acceptance, and usage of consumer rating websites such as Trip Advisor, coupled with the increasing emphasis on PPI and patient experience in the NHS, and the millions of pounds investment into OPF websites by the NHS [44], it is surprising that this study (alongside Patel et al [36]), unlike previous academic work on online rating websites, questions whether patients and carers really want or need OPF to give feedback about GPs in England.

This is because the findings indicate that although awareness is not so poor of doctor-rating websites when compared to awareness of giving feedback in general, past usage is extremely uncommon at 0.4% for feedback about GPs, and so is future consideration to use doctor-rating websites for giving feedback about GPs 82.0% of the public indicated that they will not consider using doctor-rating websites to give feedback in the future; although a further 32.9% of the population would consider using doctor-rating websites but not to leave feedback for a GP. Furthermore, only 4.0%-5.0% of those who would consider leaving feedback in the future selected doctor-rating websites as their most preferred method to leave feedback about a GP.

This, as well as the different predictors found for awareness, usage, and future consideration to use OPF websites, all appear to suggest that (1) OPF websites may not be an effective channel for collecting feedback on patient experience in general practice (and hence the NHS should provide alternative methods of collecting feedback), and (2) feedback on OPF websites is not likely to be representative of the patient experience in the near future. Although this may not be a pertinent problem for GPs and GP providers using the patient experience data for improvement (because improvement even based on 1 piece of patient feedback could potentially be useful), fundamentally it is a huge problem for the use of OPF for selection (ie, patient choice), and for monitoring. This is because the results suggest that OPF is biased because it is not representative of patient experience, and therefore patients using OPF for choice of healthcare provider are basing their choice on biased and unrepresentative data, challenging strongly the popular notion that OPF is useful for patient choice, as advocated both by academics [9,11,14] and the NHS [49,50]. Furthermore, the findings appear to contradict Greaves et al’s [14] observation of associations between NHS Choices general practice ratings and patient experience measures, thus strongly questioning the usefulness of OPF as a measure of quality in health care.

Nevertheless, the findings do suggest that OPF websites fulfill a feedback gap” for a very small number of patients, and appear to support the argument that some patients, who would not normally give feedback using other methods, would leave feedback on OPF websites. Therefore, this may suggest that OPF websites could be used to improve patient experience, as feedback can be collected from those patients who may not give feedback using other channels, as long as GP Practices were willing to use OPF for improvement purposes.

## Appendix

Multimedia Appendix 1 Questionnaire (final validated version) used in this study.

Multimedia Appendix 2 Complete set of results for the question: In which, of the following ways, if any, would you prefer to give feedback about a GP?

Multimedia Appendix 3 List of recommendations for online patient feedback website providers and owners.

Multimedia Appendix 4 List of recommendations for GPs and GP Practices.

## Abbreviations

GP: general practitioner
NHS: National Health Service
NRS: National Readership Survey
OPF: Online patient feedback
PPI: patient and public involvement
