# Supplementary material for: Public Awareness, Usage, and Predictors for the Use of Doctor Rating Websites: Cross-Sectional Study in England
Source: J Med Internet Res. 2018 Jul 25;20(7):e243. doi: 10.2196/jmir.9523 (PMC6083046; doi:10.2196/jmir.9523)
Supplement: Multimedia Appendix 1 [file jmir_v20i7e243_app1.pdf]

Multimedia Appendix 1

(Please turn to the next page)

FINAL QUESTIONNAIRE

Key to codes used in instructions
SINGLE CODE: Allow one response only
MULTI CODE: Allow multiple responses
ALLOW DK: Allow don't know (a hidden response – as not sure).
Refused is coded as **REF** (it very rarely happens)
RAN LIST: randomise the order, to reduce order bias
FIX CODES: similar items so keep these items to together
OPEN ENDED: An open ended question.
ASK ALL: Question asked to all participants, not filtered.

New Screen
INTERVIEWER: PLEASE SHOW SCREEN UNTIL OTHERWISE INSTRUCTED
DP: please underline SHOW, showscreen

In this part of the survey, you will be asked about how and why you may like to or not like to give feedback about a GP. Feedback refers to reviews, rating, comments, and complaints. This feedback could be positive or negative.

Where there is reference to "GP" in this survey, it means general practitioner. This is the doctor you see when you visit a GP surgery or local health centre.

All of the questions in this part of the survey refer to giving feedback about your experience of receiving care from GPs only and not any other healthcare professional.

New Screen

SECTION A - Awareness, history and motivation for giving feedback about GPs (if any)

ASK ALL
TY01. (A1) Before this survey, to what extent, if at all, were you aware that you could give feedback about your experience of receiving care from a GP?
SINGLE CODE, ALLOW DK

1. Yes - I was aware I could give feedback
2. No - but I assumed I could give feedback
3. No - I wasn't aware I could give feedback

IF TY01'1', THEN ASK TY02
ALL WHO ARE AWARE THEY COULD GIVE FEEDBACK
TY02. (A2) In which of the following ways, if any, did you become aware that you could give feedback about your experience of receiving care from a GP?

ALLOW DK

1. I was informed about it by a GP
2. I was informed about it by another healthcare professional
3. I was informed about it by a receptionist at a GP surgery/health centre
4. I read /saw a letter/leaflet/pamphlet/poster about it from the NHS
5. I read/saw some information about it on the NHS website
6. I read/saw information about it elsewhere on the internet
7. I read/saw/heard an advert about it
8. I saw it on a TV programme
9. A friend, family member or colleague informed me
10. Other (specify)

ASK ALL

TY03. (A3) Have you ever formally given positive or negative feedback (for example by letter, email, online, feedback form, etc.) about the care you have received from a GP?

SINGLE CODE '1', MULTI CODE '2-11', ALLOW DK

1. No
2. Yes - to a GP in my surgery/local health centre
3. Yes - to my GP surgery/local health centre
4. Yes - to a GP in another surgery/health centre
5. Yes - to another GP surgery/health centre
6. Yes - to another part of the NHS
7. Yes - through PALS (Patient Advice and Liaison Services)
8. Yes - to the Care Quality Commission
9. Yes - to an independent organisation working on behalf of the NHS to collect feedback
10. Yes - to an independent organisation able to collect feedback about a GP
11. Yes - other (specify)

IF TY03'1', THEN ASK TY04

ALL WHO HAVE NEVER FORMALLY GIVEN FEEDBACK

TY04. (A5) Why haven't you given feedback about your experience of receiving care from a GP?

INTERVIEWER PROBE: WHAT OTHER REASONS? WHAT OTHER REASONS?

OPEN END, ALLOW DK

SECTION B - Consideration of giving feedback about GPs in the future, why and when?

NEW SCREEN

The next few questions are about giving feedback about your experience of receiving care from **a GP in your surgery/local health centre.**

NEW SCREEN

ASK ALL

TY05. (B1) To what extent, if at all, would you consider giving feedback about your experience of receiving care from a GP based in your surgery/local health centre in the future?

SINGLE CODE, ALLOW DK FORWARD AND REVERSE

1. Yes - definitely
2. Yes - possibly
3. No

ASK ALL

TY06. (B4) To what extent are you more or less likely to give feedback about your experience of receiving care from a GP based in your surgery/local health centre if the experience was positive or negative, or would it make no difference either way? Would you be…?

SINGLE CODE, FORWARD AND REVERSE, FIX CODE 6 TO BOTTOM, ALLOW DK

1. A lot more likely to give feedback if your experience was negative
2. A little more likely to give feedback if your experience was negative
3. It would make no difference if the feedback was positive or negative
4. A little more likely to give feedback if your experience was positive
5. A lot more likely to give feedback if your experience was positive
6. I would not give feedback

ASK ALL

TY07. (B5) How likely or unlikely are you to do each of the following things?

SINGLE CODE, FORWARD AND REVERSE, FIX DON'T KNOW TO BOTTOM ALLOW DK

1. Very likely
2. Fairly likely
3. Neither likely nor unlikely
4. Fairly unlikely
5. Very unlikely

RAN ORDER OF STATEMENTS

1. Give unprompted feedback about your experience directly to the GP
2. Give feedback about your experience if a GP in your surgery/local health centre asked you to.

3. Give feedback about your experience if your GP surgery/local health centre asked you to.

SECTION C - Preference on mode of feedback

IF TY05'1 or 2' OR TY06'1-5'OR TY07'1-3' at COL 123, THEN ASK TY08
ALL WHO WOULD CONSIDER GIVING FEEDBACK
TY08. (C1&C2) In which of the following ways, if any, would you prefer to give **[INSERT POSITIVE/NEGATIVE]** feedback about a GP? Please choose up to three. And which would be your preferred way?

STATEMENTS - ROTATE ORDER

1. Feedback about a positive experience
2. Feedback about a negative experience

COLS- MULTI CODE COL 1 AND ALLOW MAX 3, SINGLE CODE 17, ALLOW DK AND SINGLE CODE COL 2 ALLOW DK

1. Top 3
2. Main preference

ROWS-

1. Give feedback directly to the GP (either in person or by Telephone)
2. Write a letter directly to the GP
3. Send an email directly to the GP
4. Give feedback to your GP surgery/local health centre manager (either in person or by Telephone)
5. Write a letter to your GP surgery/local health centre's manager
6. Send an email to your GP surgery/local health centre's manager
7. Fill in a feedback form at your GP surgery/local health centre (this could be anonymous or not)
8. Fill in a feedback form on the GP surgery/local health centre's website (this could be anonymous or not)
9. Post feedback on an NHS website that publishes feedback for GPs (and everyone can then read the feedback) (this could be anonymous or not)
10. Post feedback on an independent website that publishes feedback for GPs (and everyone can then read the feedback) (this could be anonymous or not)
11. Give feedback through an app on your smartphone directly to the GP surgery/local health centre (this could be anonymous or not)
12. Give feedback through an app on your smartphone, which is then published to an NHS/Independent website (this could be anonymous or not)
13. Give feedback through PALS (Patient Advice and Liaison Services)

14. Contact the Care Quality Commission
15. Give feedback on social media such as Facebook, Twitter, etc.
16. Other(specify)
17. I would not give feedback for a GP

MATCH ORDER OF ASKING TY09A AND TY09B AS TY08.
IF TY08 1_'1/16' THEN ASK TY09A
ALL WHO WERE ABLE TO SAY HOW THEY WOULD PREFER TO GIVE POSITIVE FEEDBACK
TY09A. **(C1(b) & C3)** You said you would prefer to give POSITIVE feedback in the following ways: **[INSERT CODES AT TY08_1].** Why do you say that?

INTERVIEWER PROBE: WHAT OTHER REASONS? WHAT OTHER REASONS
OPEN-ENDED, ALLOW DK

IF TY08 2_'1/16' THEN ASK TY09B
ALL WHO WERE ABLE TO SAY HOW THEY WOULD PREFER TO GIVE NEGATIVE FEEDBACK TY09B. You said you would prefer to give NEGATIVE feedback in the following ways: **[INSERT CODE AT TY08_2].** Why do you say that?

INTERVIEWER PROBE: WHAT OTHER REASONS? WHAT OTHER REASONS
OPEN-ENDED, ALLOW DK

Section D - Awareness and consideration of the use of online patient feedback

---

NEW SCREEN
ASK ALL
The next section asks about doctor rating websites. These are websites which you can use to search for health services in your local area or other areas and where patients and carers can leave anonymous feedback about their experience of using health services or receiving care from health care professionals, including doctors and GPs.

The feedback is published on the website, so people accessing the website can read and compare the reviews of the health services in their local area, or other areas.

ASK ALL
TY10. (D1) Before this survey, were you aware, or not, of doctor rating websites?
SINGLE CODE, ALLOW DK

1. Yes
2. No

IF TY10 '1', THEN ASK TY11
ALL WHO ARE AWARE OF DOCTOR RATINGS WEBSITES
TY11. (D2a) Which, if any, of the following doctor rating websites are you aware of?

MULTI CODE 1-5 RAN CODES 1-5, SINGLE CODE 6 FIX TO BOTTOM, ALLOW DK

1. www.nhs.uk/service-search
2. www.iwantgreatcare.org
3. www.patientopinion.co.uk
4. www.privatehealth.co.uk
5. Other (specify)
6. I am not aware of any specific doctor rating websites.

IF TY10 '1', THEN ASK TY12
ALL WHO ARE AWARE OF DOCTOR RATINGS WEBSITES
TY12. (D2b) In which of the following ways, if any, did you become aware of doctor rating websites?
And how did you first become aware?
GRID
COLS

1. Aware MULTI CODE, RAN LIST, FIX CODES 1-3 TOGETHER AND FIX CODE 5 AND 6 TOGETHER
2. First become aware SINGLE CODE, ORDER AS COL 1, FIX CODES 1-3 TOGETHER AND FIX CODE 5 AND 6 TOGETHER

ROWS ALLOW DK

1. I was informed about it by a GP
2. I was informed about it by another healthcare professional
3. I was informed about it by a receptionist at a GP surgery/health centre
4. I read /saw a letter/leaflet/pamphlet/poster about it from the NHS
5. I read/saw some information about it on the NHS website
6. I read/saw information about it elsewhere on the internet
7. I read/saw/heard an advert about it
8. I saw it on a TV programme
9. A friend, family member or colleague informed me
10. Other (specify)

IF TY10'1' THEN ASK TY13
ALL WHO ARE AWARE OF DOCTOR RATING WEBSITES
TY13. (D3) Have you ever used a doctor rating website, or not?
SINGLE CODE, ALLOW DK

1. Yes
2. No

IF TY13 '1', THEN ASK TY14
ALL WHO HAVE USED A DOCTOR RATING WEBSITES
TY14. (D3b) For which of the following reasons, if any, have you used a doctor rating website?

MULTI CODE, FIX CODES 4-5 TOGETHER, RAN, ALLOW DK /NONE OF THESE

1. To find a GP/doctor/consultant/hospital
2. To read GP/doctor/consultant/hospital reviews/ratings
3. To give feedback or review your experience of **the NHS**
4. To give feedback about your experience of receiving care **from a GP** in your surgery/local healthcare centre
5. To give feedback about your experience of receiving care **from a GP** <u>in another</u> surgery/local healthcare centre
6. Other (specify)

SHOWCARD TY15

IF TY14'4 or 5', THEN ASK TY15

ALL WHO HAVE LEFT FEEDBACK FOR A GP ON A DOCTOR RATING WEBSITE

TY15. (D5) Thinking about the last time you left feedback for a GP on a doctors rating website, which, if any, of these reasons motivated you to leave feedback about that GP?

MULTI CODE ALLOW DK

<u>SHOWCARD</u>

Experience

1. I wanted to let the GP know how I much appreciated the consultation
2. I wanted to let the GP know that I appreciated the quality of their service in general
3. The reception or admin staff were really helpful
4. It was easy to get an appointment
5. The service was much better than what I'm used to/have experienced in the past

Sharing the experience

6. I believe sharing my experience would benefit the GP
7. I wanted to share my experience with other people
8. I thought other people could benefit from knowing about my experience
9. I wanted to ask other people's advice about my experience
10. I believe sharing my experience would make me feel better
11. I wanted to know if other people's experiences with the GP were the same as mine
12. I wanted to alert people to the mistakes or failings of the GP

Specific issue

13. I wanted to comment on my treatment or the service in general
14. I wanted to comment on a specific consultation

To get a response

15. I wanted to prompt the local health authority to respond to a complaint I had
16. I wanted to improve the service received from the GP

So it would be taken seriously

17. I believed the GP would take my experience more seriously if I shared it online
18. I believed the GP Surgery/local health centre would take my experience more seriously if I shared it online

Negative experience

19. I didn't like the attitude of the GP
20. I didn't like the attitude of the General Practice Nurse I saw

21. I didn't like the attitude of the reception or admin staff
22. I couldn't get an appointment
23. I couldn't see a doctor during the appointment
24. I have problems using the surgery's systems

Other

25. Other specify

IF TY14'4 or 5', THEN ASK TY16

ALL WHO HAVE LEFT FEEDBACK FOR A GP ON A DOCTOR RATING WEBSITE

TY16. (D4) Thinking about the last time you gave feedback about a GP on a doctor rating website, was the feedback mainly positive, mainly negative or was it equally positive and negative?

SINGLE CODE, ROT, ALLOW DK

1. Mainly positive
2. Mainly negative
3. Equally positive and negative

ASK ALL

TY17. (D6) To what extent if at all, would you consider using a doctor rating website in the future?

SINGLE CODE, FORWARD AND REVERSE

1. Yes - definitely
2. Yes - possibly
3. No

ASK ALL

TY18. (D6a) For which of the following reasons, if any, would you consider using a doctor rating website?

MULTI CODE, FIX CODES 4-5 TOGETHER, RAN, ALLOW DK

1. To find a GP/doctor/consultant/hospital
2. To read GP/doctor/consultant/hospital reviews/ratings
3. To give feedback or review your experience of **the NHS**
4. To give feedback about your experience of receiving care **from a GP** in your surgery/local healthcare centre
5. To give feedback about your experience of receiving care **from a GP** <u>in another</u> surgery/local healthcare centre
6. Other (specify)
7. I would not use a doctor rating website for any reason

New Screen
INTERVIEWER: PLEASE DO NOT SHOW SCREEN UNTIL OTHERWISE INSTRUCTED
DP: please underline SHOW, showscreen

IF NOT TY18'4 or 5', THEN ASK TY19

ALL WHO WOULD NOT USE A DOCTOR RATING WEBSITE TO LEAVE FEEDBACK ABOUT A GP

TY19. (D9) Why wouldn't you use a doctor rating website **to give feedback** about your experience of receiving care from a GP?
INTERVIEWER PROBE FULLY: What other reasons?
ALLOW DK
OPEN-ENDED

IF TY18'4 or 5', THEN ASK TY20

TY20. (D7) Why would you consider using a doctor rating website **to give feedback** about your experience of receiving care from a GP?

INTERVIEWER: PROBE FULLY. What other reasons?

ALLOW DK

OPEN ENDED

New Screen
INTERVIEWER: PLEASE SHOW SCREEN UNTIL OTHERWISE INSTRUCTED
DP: please underline SHOW, showscreen

IF TY18'4 or 5', THEN ASK TY21

ALL WHO WOULD GIVE FEEDBACK ON A DOCTOR RATING WEBSITE

TY21. (D8a) If you were to give feedback about a GP in your surgery/local health centre on a doctor rating website, which of the following methods, if any, would you use?

And which is your preferred method?

GRID - COLS, MULTI CODE COL 1, SINGLE CODE COL 2
MASK LIST AT COL 2 - ONLY SHOW RESPONSES IN COL 1

1. Method(s) would use
2. Preferred method

ROWS - MULTI CODE, RAN, FIX OTHER TO BOTTOM, ALLOW DK

1. A computer/laptop which you have ready access to
2. A free app on your smartphone/other device
3. A web browser on your smartphone
4. An iPad or digital device available in the waiting area at your GP surgery
5. A feedback card at your GP surgery which you can write on (the feedback would then be placed online by your GP surgery)

6. Other (specify)

IF TY21_2’1/6’ THEN ASK TY22

New Screen

INTERVIEWER: PLEASE DO NOT SHOW SCREEN UNTIL OTHERWISE INSTRUCTED

DP: please underline SHOW, showscreen

TY22. (D8b) You said that you would prefer to leave feedback on a doctor rating website, using **[INSERT ANSWER AT TY21 COL2].** Why is this your preferred method?

INTERVIEWER: PROBE FULLY. What other reasons?

ALLOW DK

1. Easy to use
2. Convenient
3. Quicker- feedback appears online immediately
4. Can provide feedback at the time of the issue
5. Can provide feedback later
6. Can provide feedback at any time
7. Don’t have access to the internet/computer/mobile phone
8. Don’t know how to use internet/apps/ipads
9. Don’t trust the GP surgery to add my feedback online
10. Concerns about anonymity if provide feedback on paper
11. Prefer to use my own device- don’t trust NHS IT systems
12. Other specify

SECTION F - Anonymity and privacy

IF TY05’1 or 2’ OR TY17’1 or 2’, THEN ASK TY23

ASK ALL WHO WOULD CONSIDER GIVING FEEDBACK

TY23. (F1a & F2a & F3a & F4a) I’m now going to show you a list of things people may choose to do when giving feedback for a GP in their surgery/local health centre. For each one I’d like you to tell me in which situation, if any, you would choose to do each one.

STATEMENTS:

SHOW STATEMENTS 1&3 IF TY05’1 or 2’; SHOW STATEMENTS 2&4 IF TY17’1 or 2’

1. Give your full name on feedback you give directly to your GP surgery/local health centre
2. Give your full name on feedback you leave on a doctor rating website
3. Mention a GP by name on feedback you give to your GP surgery/local health centre
4. Mention a GP by name on feedback you leave on a doctor rating website

MULTI CODE 1-3, SINGLE CODE 4-5, ROT CODES 1-2, ALLOW DK

1. When giving feedback about positive experiences
2. When giving feedback about negative experiences
3. Other (specify)
4. I would not do this
5. I would not give feedback about a GP using this method

SECTION G - Other factors that may be affecting patient intention to give feedback about GPs

ASK ALL

TY24. (G4) I'm now going to show you some statements **<u>about leaving feedback for GPs</u>**. Please tell me to what extent you agree or disagree with each one.

RANDOMISE ORDER OF STATEMENTS

STATEMENTS

1. Giving/leaving feedback about a GP is something I have thought about before
2. I would leave feedback because I want someone to improve the service so that it can be better for the next patient
3. Other people could benefit from knowing about my experience of care from a GP
4. I am concerned that leaving feedback with my full name on it will impact my relationship with a GP
5. Leaving feedback for GPs will make no difference
6. I would be concerned about my privacy when leaving feedback for a GP on a feedback form at the GP surgery/health centre
7. I would be concerned about my privacy when leaving feedback for a GP online on a doctor rating website
8. GPs do not want patients' feedback
9. I do not know how or where to leave feedback for a GP
10. I would benefit from reading about other peoples' experiences of receiving care from a GP
11. I would find it easier to give feedback online on a doctor rating website rather than giving it on a feedback form at the GP surgery/health centre
12. I would consider leaving feedback about a GP on social media (such as Facebook or Twitter)
13. I would prefer to leave feedback for a GP online on a doctor rating website rather than leave it on a feedback card at the GP surgery/health centre
14. Sharing my experience of receiving care from a GP online on a doctor rating website would make me feel better
15. Sharing my experience online on a doctor rating website would be taken more seriously by the GP or the GP practice

SINGLE CODE, FORWARD AND REVERSE, ALLOW DK

1. Strongly agree
2. Tend to agree
3. Neither agree nor disagree
4. Tend to disagree
5. Strongly disagree

ASK ALL

TY25. (G3) Approximately how many GPs are there in your current GP surgery/ local health centre?

SINGLE CODE, ALLOW DK

1. 1 GP
2. 2-3 GPs
3. 4-5 GPs
4. 6-9 GPs
5. More than 10 GPs

ASK ALL

TY25. (G2) Have you ever used the Internet to search for health information?

SINGLE CODE, ALLOW DK

1. Yes
2. No

ASK ALL

TY27. (G1) Do you have a long term health condition? A long term health condition can be described as a condition that cannot be cured, at present, but can be controlled by medication or other therapies. Long term health conditions include (but are not limited to) conditions such as diabetes, heart disease, high blood pressure, emphysema, asthma, arthritis, depression, dementia, etc.

SINGLE CODE

1. Yes
2. No
3. Don't Know
4. Prefer not to say

SECTION H - Socio-demographics

ASK ALL (These standard demographic questions below were devised by IPSOS Mori are asked to all participants)

Question REGION

Standard region (from sample point)

1. North
2. North-West
3. Yorks & Humberside
4. West Midlands
5. East Midlands
6. East Anglia
7. South West
8. South East
9. Greater London
10. Wales
11. Scotland

Question SEX

Sex of respondent

1. Male
2. Female

Question AGE Numeric.

AGE OF RESPONDENT: Enter exact age

<OPEN ENDED>
Refused

Question QUAL

Using this card, please tell me which, if any, is the highest educational or professional qualification you have obtained. Just read out the letter or letters which apply.
(IF STILL STUDYING, CHECK FOR HIGHEST ACHIEVED SO FAR)

1. A GCSE/O-Level/CSE
2. B Vocational qualifications (=NVQ1+2)
3. C A-Level or equivalent (=NVQ3)
4. D Bachelor Degree or equivalent (=NVQ4)
5. E Masters/PhD or equivalent
6. F Other
7. G No formal qualifications
8. H Still studying

   Don't Know

Question CIEWORKSTATUS

Could you please tell me which of these applies to the chief income earner?

1. Have paid job - Full time (30+ hours per week)
2. Have paid job - Part time (8-29 hours per week)
3. Have paid job - Part time (Under 8 hours per week)
4. Not working - Housewife
5. Self-employed
6. Full time student
7. Still at school
8. Unemployed and seeking work
9. Retired
10. Not in paid work for other reason
11. Not in paid work because of long term illness or disability

Refused

Question SOCG

WORK STATUS : %SOCTX%
DETAILS OF CIE: %COPIED%
CODE IN SOCIAL GRADE:

1. A
2. B
3. C1
4. C2
5. D
6. E

HH-DURA Question

Which, if any, of these items are there in your household?
PROBE: Which others? INTERVIEWER: MOBILE PHONE DOES NOT INCLUDE CAR PHONE.
DURABLES
Entertainment
D. A smart/connected TV set that is connected to the internet (a TV set that is connected directly to the internet and not through another device such as a games console, computer or set top box)
L. A 3D (3 dimensional) TV set (a TV that allows you to watch movies and TV shows in 3D, as long as you are wearing special 3D glasses when you watch it)
P. An HDTV set (TV can receive High Definition television that has a significantly higher resolution than traditional formats)
G . Any Other TV
V. DVD player / Blu-ray player
N. Satellite or Cable Subscription (i.e. a monthly subscription you pay to watch extra channels)
U. Freeview or Freesat (i.e. digital TV programmes accessed through a set-top box or satellite dish, that you do not have to pay a subscription for)H. Personal Video Recorder (PVR) / DTR - e.g. Sky+ / V+ / Freeview+ / an in-built hard-drive on your TV or set-top box
Q. Last generation games console (NOT home computer) (e.g. Wii, PS3, Xbox 360)

A. Next generation games console (NOT home computer) (e.g. Wii U, PS4, Xbox One)
X. A handheld games console (e.g. Nintendo DS, PS Vita)
F. MP3 Player (e.g. Apple Ipod or Sony Walkman)
K. Other Games console
Miscellaneous
R. Personal Computer / Desktop PC (PC, Mac or other type of home computer)
B. Laptop (PC/Apple, including netbooks)
S. A tablet (e.g. an iPad, Samsung Galaxy Tab or Amazon Kindle Fire)
I. An eBook reader (e.g. Amazon Kindle/Sony Reader)
E. Simple/feature mobile phone (a phone with simple features and limited media / internet functions)
W. Smart phone (e.g. iPhone, Blackberry, Android, Windows)
M. Telephone (landline, i.e. NOT a mobile phone)
Cards
O. Debit card/s (e.g. Maestro, Visa Debit Card)
T. Credit card/s (e.g. Visa Credit Card, Mastercard, American Express)
C. Storecard (A card that can typically on be used to buy items or services in one brand of stores)
J. Loyalty card - (A card that typically provides discounts or rewards within store)
Refused
Don't know
None of these

Question NETFQ

Which of these best describes your use of the internet? Please include all use of the internet, including sending and receiving emails

1. Several times a day
2. Around once a day
3. 4 or 5 times a week
4. 2 or 3 times a week
5. Around once a week
6. 2 or 3 times a month
7. Around once a month
8. Less than around once a month
9. Never but I have access
10. Never but I do not have access

Question DAYOF

INTERVIEWER - CODE DAY OF WEEK
Monday
Tuesday
Wednesday
Thursday
Friday
Saturday

Sunday

Question INCOME

Could you please give me the letter from this card for the group in which you would place your total household income per year from all sources, before tax and other deductions?

WEEKLY INCOME		ANNUAL INCOME	
	£		£
E.	Less than 86	E.	Up to 4,499
K.	87 - 124	K.	4,500 - 6,499
S.	125 - 144	S.	6,500 - 7,499
P.	145 - 182	P.	7,500 - 9,499
L.	183 - 221	L.	9,500 - 11,499
J.	222 - 259	J.	11,500 - 13,499
A.	260 – 298	A.	13,500 - 15,499
T.	299 - 336	T.	15,500 - 17,499
D.	337 - 480	D.	17,500 - 24,999
N.	481 - 576	N.	25,000 - 29,999
C.	577 - 769	C.	30,000 - 39,999
F.	770 – 961	F.	40,000 - 49,999
R.	962 - 1442	R.	50,000 - 74,999
H.	1443 - 1923	H.	75,000 - 99,999
M.	1924 or more	M.	100,000 or more

Don't know
Refused

Question ETHNICITY

Which group on this card do you consider you belong to?
Please read out the letter.

A. White - English / Welsh / Scottish / Northern Irish / British
B. White - Irish
C. White - Gypsy or Irish Traveller
D. White - Any other White background
E. Mixed - White and Black Caribbean
F. Mixed - White and Black African
G. Mixed - White and Asian
H. Mixed - Any other Mixed / multiple ethnic background
I. Asian/Asian British - Indian
J. Asian/Asian British - Pakistani
K. Asian/Asian British - Bangladeshi
L. Asian/Asian British - Chinese
M. Asian/Asian British - Any other Asian background
N. Black - African
O. Black - Caribbean

P. Black - Any other Black / African / Caribbean background
Q. Arab
R. Any other ethnic group
Don't know
Refused
