# Supplementary material for: Public Awareness, Usage, and Predictors for the Use of Doctor Rating Websites: Cross-Sectional Study in England
Source: J Med Internet Res. 2018 Jul 25;20(7):e243. doi: 10.2196/jmir.9523 (PMC6083046; doi:10.2196/jmir.9523)
Supplement: Multimedia Appendix 2 [file jmir_v20i7e243_app2.pdf]

Multimedia Appendix 2

Table

TY08 Summary: In which, of the following ways, if any, would you prefer to give feedback about a GP?

Base: All Adults 15+ in England who would consider giving feedback about a GP

	Positive		Negative	
	One of top 3 preferred ways to give feedback (n, %)	Most preferred way to give feedback (n, %)	One of least 3 preferred ways to give feedback (n, %)	Least preferred way to give feedback (n, %)
**Unweighted Base** (total n)	777	777	777	777
**Weighted Base** (total n)	776	776	776	776
**Give feedback directly to the GP (either in person or by Telephone)**	360	240	292	184
	46%	*31%*	*38%*	*24%*
**Write a letter directly to the GP**	179	63	186	77
	23%	*8%*	*24%*	*10%*
**Send an email directly to the GP**	217	94	196	87
	28%	*12%*	*25%*	*11%*
**Give feedback to your GP surgery/ local health centre manager (either in person or by Telephone)**	103	36	131	52
	13%	*5%*	*17%*	*7%*
**Write a letter to your GP surgery/ local health centre's manager**	59	17	89	35
	8%	*2%*	*12%*	*5%*
**Send an email to your GP surgery/ local health centre's manager**	79	31	118	35
	10%	*4%*	*15%*	*5%*
**Fill in a feedback form at your GP surgery/local health centre (this could be anonymous or not)**	178	72	181	79
	23%	*9%*	*23%*	*10%*
**Fill in a feedback form on the GP surgery/local health centre's website (this could be anonymous or not)**	103	43	132	51
	13%	*6%*	*17%*	*7%*
**Post feedback on an NHS website that publishes feedback for GPs (and everyone can then read the feedback) (this could be anonymous or not)**	64	24	67	25
	8%	*3%*	*9%*	*3%*
**Post feedback on an independent website that publishes feedback for GPs (and everyone can then read the feedback) (this could be anonymous or not)**	31	10	34	10
	4%	*1%*	*4%*	*1%*

**Give feedback through an app on your smartphone directly to the GP surgery/local health centre (this could be anonymous or not)**	34	17	49	23
	4%	*2%*	*6%*	*3%*
**Give feedback through an app on your smartphone, which is then published to an NHS/Independent website (this could be anonymous or not)**	*35*	*12*	*33*	*9*
	5%	*1%*	*4%*	*1%*
**Give feedback through PALS (Patient Advice and Liaison Services)**	*25*	*5*	*30*	*4*
	3%	*1%*	*4%*	*1%*
**Contact the Care Quality Commission**	*12*	*2*	*22*	*2*
	2%	*	*3%*	*
**Give feedback on social media such as Facebook, Twitter, etc.**	*19*	*6*	*26*	*5*
	3%	*1%*	*3%*	*1%*
**Online**	*1*	*1*	*1*	*1*
	*	*	*	*
**Fill in a questionnaire by post**	*1*	*	*	*
	*	*	*	*
**Speak to the receptionist**	-	-	*1*	*1*
	-	-	*	*
**Other**	*2*	*1*	*1*	-
	*	*	*	-
**I would not give feedback for a GP**	*96*	*96*	*80*	*80*
	12%	*12%*	*10%*	*10%*
**No answer**	*1*	*1*	*4*	*5*
	*	*	*	*1%*
**Don't know**	*5*	*5*	*7*	*7*
	1%	*1%*	*1%*	*1%*
**NET: FEEDBACK DIRECTLY TO GP**	***533***	***397***	***486***	***348***
	69%	***51%***	***63%***	***45%***
**NET: FEEDBACK TO GP SURGERY/LOCAL HEALTH CENTRE MANAGER**	***219***	***84***	***296***	***123***
	28%	***11%***	***38%***	***16%***
**NET: FILL IN FEEDBACK FORM**	***246***	***115***	***279***	***130***
	32%	***15%***	***36%***	***17%***
**NET: POST FEEDBACK ON A WEBSITE**	***89***	***33***	***90***	***36***
	12%	***4%***	***12%***	***5%***
**NET: FEEDBACK VIA AN APP**	***58***	***29***	***70***	***33***
	7%	***4%***	***9%***	***4%***
