# Supplementary material for: Public Awareness, Usage, and Predictors for the Use of Doctor Rating Websites: Cross-Sectional Study in England
Source: J Med Internet Res. 2018 Jul 25;20(7):e243. doi: 10.2196/jmir.9523 (PMC6083046; doi:10.2196/jmir.9523)
Supplement: Multimedia Appendix 3 [file jmir_v20i7e243_app3.pdf]

List of recommendations for online patient feedback website providers and owners.

**RECOMMENDATIONS FOR ONLINE PATIENT FEEDBACK (OPF) WEBSITE PROVIDERS/OWNERS**	
**A. MAKE SYSTEMATIC CHANGES TO OPF WEBSITES**	
1	Allow patients to leave feedback on OPF websites, but when the feedback is presented to other patients for “choice”, it must be part of a collection of measures including patient feedback collected using other methods, and other measures such as the clinical competency of the GP, findings from the CQC report, and safety results. All of these must also be combined with the overall “star rating” that is displayed for each GP or GP practice. Patients cannot exercise “choice” accurately based alone on patient feedback left on OPF websites.
**B. POSITIVELY PROMOTE AND CONVINCE PATIENTS AND GPS ABOUT THE VALUE OF OPF**	
2	Use targeted marketing material (or provide information) both on the website and elsewhere to positively promote OPF websites to patients and carers.
3	Make the public aware of OPF websites, because very few patients were aware of specific OPF websites. A national campaign to promote OPF websites will help increase the number of patients and types of patients leaving feedback and reviews, and therefore the feedback left online is less likely to be biased and unrepresentative. This will mean patients will be able to make a valid “choice” and may also mean that GPs will take OPF more seriously. The promotion could be conducted through traditional marketing routes through GP practices, as well as digital methods, such as social media, and TV ads.
4	Marketing material or techniques used could be focused on those who are less or more likely to leave feedback about GPs. Those that are more likely include: (1) females; those between the ages of 35-44, 55-59, and 60-64, (2) those who have long-term health conditions, (3) those who have used the internet in the past to search for health information, and (4) those with higher qualifications.
**C. MAKE OPF WEBSITES ACCESSIBLE TO ALL PATIENTS**	
5	The website should be designed so that it is easy and straightforward to use (user friendly), and easily accessible. This includes ensuring that the website is easy to use with all smartphone devices and is also disability and age friendly [44].
6	Provide optional alternative methods and modes to leave feedback on OPF websites:
7	Provide alternative modes to giving feedback on OPF websites, such as paper feedback forms at the GP surgery that could be used by patients, posted to OPF website providers, or given back to the GP surgery and then be placed online. This would be especially useful for those patients who do not have internet access or cannot use a website but are happy to share their feedback online, or those who want to give feedback whilst they are situated in the practice, but do not have a smartphone or access to the internet.
8	Consider providing an option on the website to directly contact the GP. For example, the option to email the GP from the OPF website if the GP has consented to that, or the option to leave feedback on an OPF website that will not be published but would be forwarded on to the GP or the GP practice.
9	Current evidence does not suggest that creating a separate app to leave feedback on doctor rating websites will rapidly increase usage. However, if patients are already using an app to book appointments for example, consider integrating a feedback function on to that.
10	Kiosks or iPads could be provided to patients at the GP surgery as an alternative method to leave feedback. This may encourage a few patients to give feedback, and it is something OPF website providers should explore further with GP practices and patients.
11	There is no need currently to invest in social media as a platform for collecting patient feedback about GPs. This may however change in the future.
