# Supplementary material for: Public Awareness, Usage, and Predictors for the Use of Doctor Rating Websites: Cross-Sectional Study in England
Source: J Med Internet Res. 2018 Jul 25;20(7):e243. doi: 10.2196/jmir.9523 (PMC6083046; doi:10.2196/jmir.9523)
Supplement: Multimedia Appendix 4 [file jmir_v20i7e243_app4.pdf]

List of recommendations for GPs and GP Practices.

**RECOMMENDATIONS FOR GPS AND GP PRACTICES**	
**A. GENERAL RECOMMENDATIONS RELATED TO COLLECTING PATIENT FEEDBACK**	
Promote giving feedback about GPs to patients:	
1	Make patients and carers aware that they can leave feedback about and for GPs. The material or techniques used for marketing could focus on those who are more or less likely to give feedback about GPs. The following are more likely to leave feedback: (1) females between the ages of 35-44, 55-59, and 60-64, (2) those who have long term health conditions, (3) those who have used the internet in the past to search for health information, and (4) those with higher qualifications.
Examine the methods provided to patients to give feedback:	
2	There is no one feedback method that is preferred by the majority of patients, therefore the GP/practice should consider providing a few methods of feedback, based on consultations with their patients.
3	The methods that GPs and GP practices provide for patients to leave feedback about a GP need to be easy and convenient to use.
4	Where possible, provide direct method/s for patients to give feedback about GPs, including the option to email the GP directly. This is because half of those who would consider giving feedback about GPs prefer to give it directly to the GP, and email was preferred by more patients than writing a letter.
5	Consider alternatives to the NHS Friends and Family Test (FFT) card to get feedback from patients (because only 10% of patients who would consider leaving feedback selected paper-based feedback as their most preferred method to leave negative feedback about GPs).
6	There is no need currently to invest in social media as a platform for collecting patient feedback about GPs. This may however change in the future.
**B. RECOMMENDATIONS SPECIFIC TO ONLINE PATIENT FEEDBACK (OPF) WEBSITES (OR DOCTOR RATING WEBSITES)**	
7	To increase the number of people giving feedback on OPF websites, the GP/practice needs to: (1) make patients aware that such websites exist, (2) convince them that they are secure and easy to use [44], (3) convince them that the feedback will be useful to GPs and to other patients [44], and (4) and convince them that the GP will read and use the feedback for improvement [44]. Feedback cards should also be provided at the surgery that patients can use to write feedback with a pen, which can then be uploaded online.
8	Alternatively, if GPs/practices do not want patients to use OPF websites, they should provide clear alternative ways (digital and non-digital) for patients to give feedback about GPs, including direct ways to leave feedback, such as an email address for the practice/GP, or an easy to use private feedback form on the GP practice website where patients can leave feedback anonymously.
9	If some of the patients are already using an app to make appointments for example, consider integrating a feedback function on that app.
10	Kiosks or iPads could be provided to patients at the surgery to encourage patients to give feedback. However, evidence suggests that it will not dramatically increase usage of OPF websites.
11	If one of the aims is to increase patient choice by collecting patient feedback, GP practices could collect patient feedback using non-online methods, and then place that feedback online, which other patients can then use to choose a provider.
